# The distributive and structural characteristics of bronchus-associated lymphoid tissue (BALT) in Bactrian camels (*Camelus bactrianus*)

**DOI:** 10.7717/peerj.6571

**Published:** 2019-03-11

**Authors:** Wanhong He, Wangdong Zhang, Cuicui Cheng, Jianfei Li, Xiuping Wu, Min Li, Zhihua Chen, Wenhui Wang

**Affiliations:** College of Veterinary Medicine, Gansu Agricultural University, Lanzhou, Gansu, China

**Keywords:** Bactrian camels, Bronchial tree, Bronchus-associated lymphoid tissue (BALT), Distribution characteristics

## Abstract

**Background:**

Bronchus-associated lymphoid tissue (BALT), distributed in the bronchial mucosa, plays a critical role in maintaining the mucosal immune homeostasis of the lower respiratory tract. The bronchial tree is a functional structure for gas exchange with the outside environment and maintains basic lung morphology.

**Methods:**

To explore the structural and distributive characteristics of BALT in Bactrian camels, twelve healthy adult Bactrian camels were divided into two groups (six in each group). The lungs, bronchial tree and BALT were observed and analysed systematically through anatomical and histological methods.

**Results:**

The results showed that Bactrian camel lungs were constituted by the left cranial lobe, left caudal lobe, right cranial lobe, right caudal lobe and accessory lobe, but lacked the middle lobe. The cranial lobe was narrow and small, the caudal lobe was extremely developed (almost four times the cranial lobe in size), and the accessory lobe was smaller than the cranial lobe; the bronchial tree, an unequal dichotomy with a tracheobronchial branch, was composed of dorsal, ventral, lateral and medial bronchiole systems. Isolated lymphoid follicles (the chief type) and aggregates of lymphoid follicles revealed two types of BALT, and germinal centres, follicle-associated epithelium and high endothelial venules could be observed in some well-developed BALT. Additionally, BALT was scattered along the bronchial tree in the entire lung, and the density increased from the trachea to the lower graded branches (densest in the bronchioles) and then decreased, with the occasional location around respiratory bronchioles or among the pulmonary mesenchyme. In the conducting portion, BALT was primarily located in the mucosa lamina propria but was also found in the submucosa, under the muscular layer, and around the submucosal glands and cartilage.

**Conclusion:**

The results demonstrated that the lung morphology of Bactrian camels was similar to that of horses, but the bronchial branches were more closely related to those of ruminants. These characteristics were in accordance with the morphological and structural variation regularity of lungs with species evolution. BALT was mainly scattered in the conducting portion, and bronchioles, as the final “checkpoint” in the surveillance, capture and recognition of antigens before pulmonary exchange, were the pivotal locational position of BALT. However, BALT at different depths of the bronchial wall of the conducting portion might be at different developmental stages. Our study provided evidence for further insight into the mucosal immunomodulatory mechanism of BALT in the respiratory system of Bactrian camels.

## Introduction

Because they directly communicate with the outside environment, the lungs, an important organ for gas exchange, are a crucial site for various types of pathogen invasion, such as influenza virus, measles virus, *Mycobacterium tuberculosis* and others. As an important constituent of mucosa-associated lymphoid tissue (MALT), bronchus-associated lymphoid tissue (BALT) plays a crucial role in maintaining and regulating lung mucosal immune homeostasis.

However, compared with gut-associated lymphoid tissue (GALT), BALT is relatively special in its development because it is not pre-programmed embryonically, and it occurs postnatally by antigen stimulation (Foo & Phipps, 2010; Pabst & Tschernig, 2010). Both BALT and GALT consist mainly of lymphocytes, dendritic cells, stroma cells, follicular dendritic cells and high endothelial venules (HEVs) (Matsuura, Matsuoka & Fuse, 1992; Moyron-Quiroz et al., 2004; Xu et al., 2003). Additionally, microfold (M) cells exist in the dome epithelium of some BALT (Pabst & Tschernig, 2010; Xu et al., 2003). Importantly, BALT is involved in the immune response (Bienenstock, 1980; Iwata & Sato, 1991) and is principally induced to produce IgA^+^ cells that secrete polymeric immunoglobulin A (pIgA). When pIgA is transported to the lumen, it induces secretory immunoglobulin A (SIgA) formation, which begins to regulate microbial homeostasis (Peterson et al., 2007), induce immune tolerance (Hapfelmeier et al., 2010; Mosha & Scheutz, 2002; Suzuki et al., 2004), inhibit inflammation (Macpherson & Uhr, 2004) and promote immune clearance (Corthesy, 2013; Phalipon et al., 2002; Strugnell & Wijburg, 2010).

Interestingly, BALT varies in different species and different physiological states of the same species. For example, BALT is regularly found in all healthy rabbits and rats (100%) and in some pigs (30%–50%) but is absent in dogs and cats (Bienenstock & McDermott, 2005; Foo & Phipps, 2010). In humans, BALT exists in only 40% of healthy adolescents and children but can be induced by infection, chronic inflammation or autoimmune disease in adults (Aloisi & Pujol-Borrell, 2006; Foo & Phipps, 2010; Halle et al., 2009; Pabst & Tschernig, 2010; Tschernig & Pabst, 2009) and by prenatal amniotic infection in foetuses and infants (Gould & Isaacson, 1993). BALT features in mice are the same as those in humans. Furthermore, the occurrence, size, and amount of BALT depend on the type and duration of antigenic exposure (Hwang, Randall & Silva-Sanchez, 2016).

Camels are different from other livestock (i.e., cattle, sheep and horses) (Zhao, 1995). Bactrian camels are an important economic livestock in northwest China and have some special biological characteristics, such as hunger, thirst, and heat and cold resistances (Zhao, Zhang & Rong, 2008; Zhou & Luo, 1990). Beyond these characteristics, the mucosal immune system of the Bactrian camel is especially distinct. For instance, there is a unique aggregated lymphoid nodule area (ALNA) in the abomasum (Wang, 2003); nodular, faviform and scrotiform Peyer’s patches (PPs) in the intestinal tract (Qi et al., 2011; Zhaxi et al., 2014); and IgG2 and IgG3 are special heavy chain antibodies (naturally devoid of light chain) (Tillib, 2011). However, there is still no report on Bactrian camels describing BALT as a part of MALT. Therefore, using anatomical and histological methods, the morphological distribution characteristics of BALT in the conducting and respiratory portions of Bactrian camel lungs were systematically analysed. The results of this study provide support for future studies exploring the mucosal immunomodulatory characteristics of BALT in the lower respiratory tract of Bactrian camels.

## Materials & Methods

### Experimental animals and group divisions

All experimental procedures were approved by the Animal Ethical and Welfare Committee of the College of Veterinary Medicine of Gansu Agricultural University (Approval No. GSAU-AEW-2016-0010). The healthy animals (Bactrian camels with disease records were all excluded according to the historical data of the experimental animals investigated) were provided from the slaughterhouse and did not starve before slaughter.

Twelve healthy adult Alashan Bactrian camels (2 to 7 years old, six males and six females) were divided into two groups: the lung-conducting portion group and the respiratory portion group, with six camels (three males and three females) in each group. Camels were anaesthetised intravenously with sodium pentobarbital (20 mg/kg) and then exsanguinated until death.

### Lung fixation

After the camels were euthanized, the chest was quickly opened, and the lungs were immediately obtained by clamping the main pulmonary artery with a snare and rapidly irrigating 10% neutral formalin into the trachea (above the cranial lobe of the lung) at 30 cm of water pressure. After stopping the flow, the trachea was ligated, and the whole lung was vertically immersed in a barrel with the same fixative for approximately one month.

### Lung respiratory portion group

**Figure 1:**
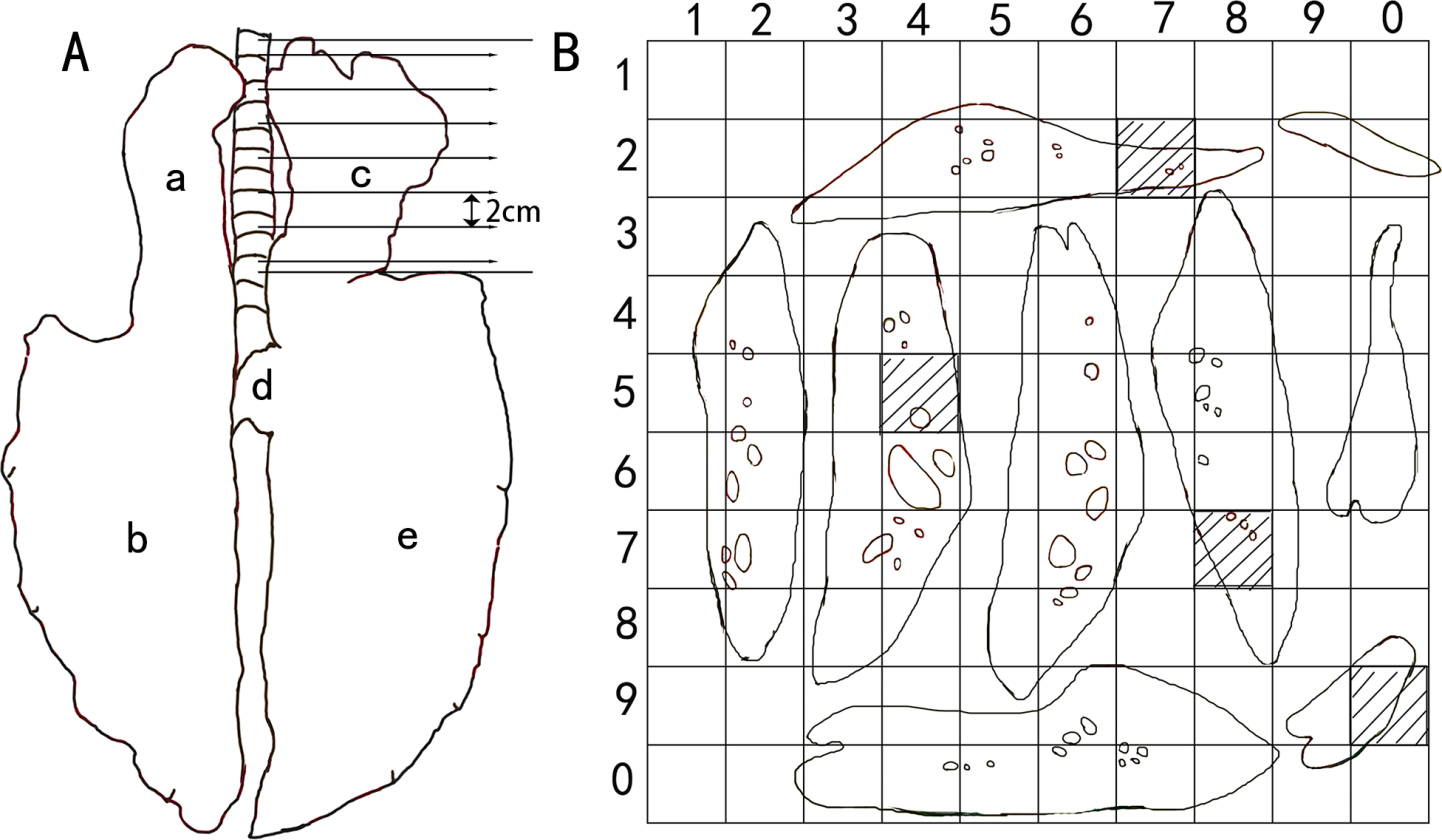
Stratified uniform random sampling (StURS) of Bactrian camel lungs. The camel lung consisted of the left cranial lobe (a), left caudal lobe (b), right cranial lobe (c), right accessory lobe (d) and right caudal lobe (e). According to these anatomical structural characteristics of the camel lung, the lung lobes were separated, and each lobe was perpendicularly cut into equal thick parallel stratum along the long axis of the lung lobe. For example, if the long axis of the right cranial lobe was approximately 21 cm, this lobe would be divided into 10 equal parts of 2 cm (A). These equal parts with an upper cut surface were randomly overlaid on a paperboard printed with a 10 × 10 grid (each side was 2 cm), and the lung tissue that hit the grid (slash squares) was randomly sampled (B). Image by Wanhong He.

According to the method of stratified uniform random sampling (StURS) (Hsia et al., 2010; Yang, 2012), in the first group, the left cranial lobe, left caudal lobe, right cranial lobe, right caudal lobe and accessory lobe were separated in each lung, and each lobe was then perpendicularly cut into equal thick parallel stratum along the long axis of each lobe. For example, if the long axis of the right cranial lobe was approximately 21 cm, this lobe would be divided into 10 equal parts of 2 cm (Fig. 1A). These equal parts with upper cut surfaces were randomly overlaid on a paperboard with a printed 10 × 10 grid (each side was 2 cm), and lung tissue that hit the grid (slash squares) was randomly sampled (Fig. 1B). Finally, four sample blocks in the left cranial lobe, six in the left caudal lobe, four in the right cranial lobe, six in the right caudal lobe and two in the accessory lobe were randomly extracted to make paraffin sections (4 µm) for each lung. These sections were stained with haematoxylin and eosin (H & E).

### Lung conducting portion group

**Figure 2:**
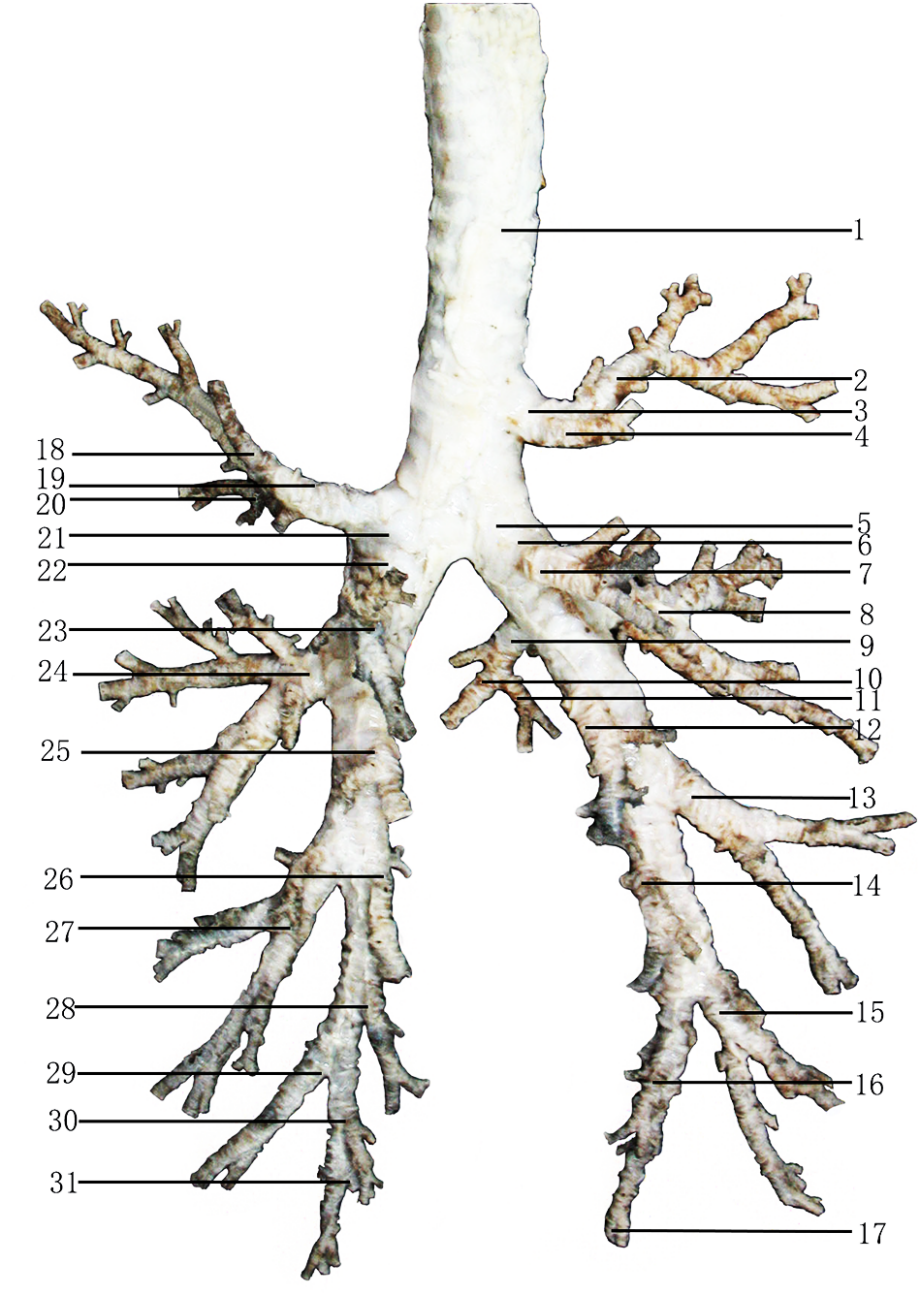
Sampling locations of the bronchial tree in Bactrian camels. 1, Trachea; 2, Cranial segmental bronchi of the right cranial lobe (Acr); 3, Lobar bronchi of the right cranial lobe; 4, Caudal segmental bronchi of the right cranial lobe (Aca); 5, Right main bronchi; 6, Lobar bronchi of the right caudal lobe; 7, First dorsal segmental bronchi of the right caudal lobe (D1); 8, First lateral segmental bronchi of the right caudal lobe (L1); 9, Lobar bronchi of the accessory lobe; 10, Ventral segmental bronchi of the accessory lobe (Acv); 11, Dorsal segmental bronchi of the accessory lobe (Acd); 12, Second dorsal segmental bronchi of the right caudal lobe (D2); 13, Second lateral segmental bronchi of the right caudal lobe (L2); 14, Fourth dorsal segmental bronchi of the right caudal lobe (D4); 15, Third lateral segmental bronchi of the right caudal lobe (L3); 16, Fifth dorsal segmental bronchi of the right caudal lobe (D5); 17, Sixth dorsal segmental bronchi of the right caudal lobe (D6); 18, Cranial segmental bronchi of the left cranial lobe (Acr); 19, Lobar bronchi of the left cranial lobe; 20, Caudal segmental bronchi of the left cranial lobe (Aca); 21, Left main bronchi; 22, Lobar bronchi of the left caudal lobe; 23, First dorsal segmental bronchi of the left caudal lobe (D1); 24, First lateral segmental bronchi of the left caudal lobe (L1); 25, Second dorsal segmental bronchi of the left caudal lobe (D2); 26, Third dorsal segmental bronchi of the left caudal lobe (D3); 27, Second lateral segmental bronchi of the left caudal lobe (L2); 28, Fourth dorsal segmental bronchi of the left caudal lobe (D4); 29, Third lateral segmental bronchi of the left caudal lobe (L3); 30, Fifth dorsal segmental bronchi of the left caudal lobe (D5); 31, Sixth dorsal segmental bronchi of the left caudal lobe (D6). Photograph by Wanhong He.

The other six lungs in the second group were slowly isolated with ophthalmic scissors and tweezers, and pictures were taken. The bronchial tree is composed of dorsal, ventral, medial and lateral bronchiole systems; therefore, the sampling locations were decided based on the anatomical features of the bronchial tree (pointed in Fig. 2). Then, samples from the trachea to the bronchiole in each bronchiole system were taken and made into paraffin sections (4 µm). However, samples of the smaller medial and ventral bronchiole systems were directly taken and made into paraffin sections (4 µm) without marking (Fig. 2). All sections were stained with H & E and periodic acid Schiff (PAS).

### Light microscopy observation and analysis

Using optical microscopy, in each group, the histological characteristics of the bronchial tree, including the dorsal, ventral, medial and lateral bronchiole systems, and the respiratory portion in each lung lobe, were carefully observed. The morphological distribution characteristics of BALT in the conducting portions of the dorsal, ventral, medial and lateral bronchiole systems and in the respiratory portion of each lung lobe were systematically observed.

## Results

### The anatomical characteristics of lungs and bronchial branches in Bactrian camels

Bactrian camel lung was approximately 4.5 kg, with a dark-red colour, and volume differences between the left lung and right lung were not obvious. Moreover, the camel lungs were composed of a left cranial lobe, left caudal lobe, right cranial lobe, right caudal lobe and accessory lobe. The cranial lobe was narrow and small, the caudal lobe was extremely developed (almost four times the cranial lobe in size), and the accessory lobe was smaller than the cranial lobe.

**Figure 3:**
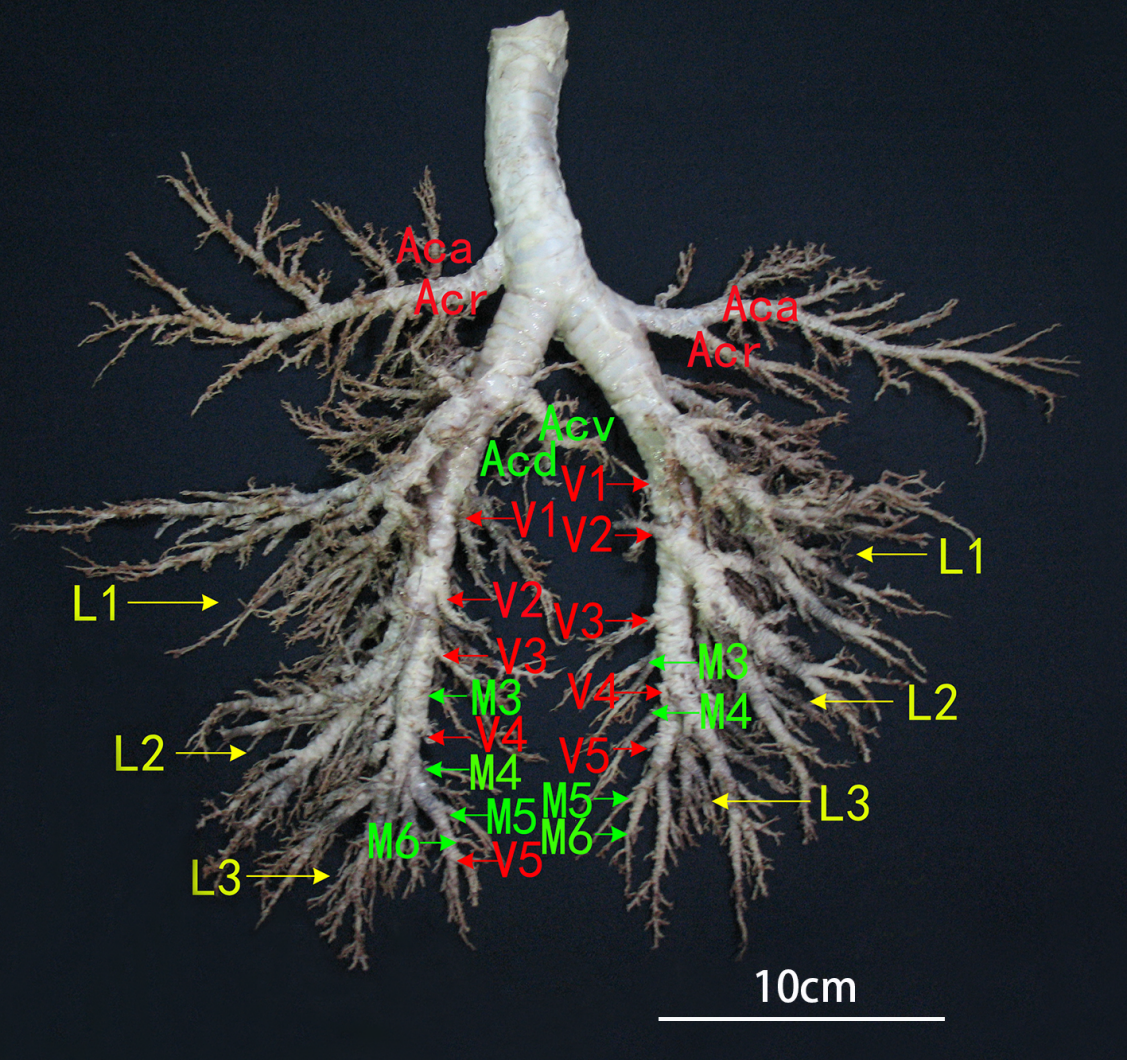
Ventral view of the bronchial tree of Bactrian camels. The bronchial tree had unequal dichotomy, and approximately three cartilaginous rings were over the top of the tracheal bifurcation; the trachea branched off into a tracheobronchial branch to form the right cranial lobar bronchi, but the left cranial lobar bronchi was the first branch of the left main bronchi. Aca, Caudal segmental bronchi of the cranial lobe; Acr, Cranial segmental bronchi of the cranial lobe; Acv, Ventral segmental bronchi of the accessory lobe; Acd, Dorsal segmental bronchi of the accessory lobe; L1–L3, Lateral segmental bronchi from the first to third segment of the caudal lobe; V1–V5, Ventral segmental bronchi from the first to fifth segment of the caudal lobe; M3–M6, Medial segmental bronchi from third to sixth segment of the caudal lobe. Photograph by Wanhong He.

**Figure 4:**
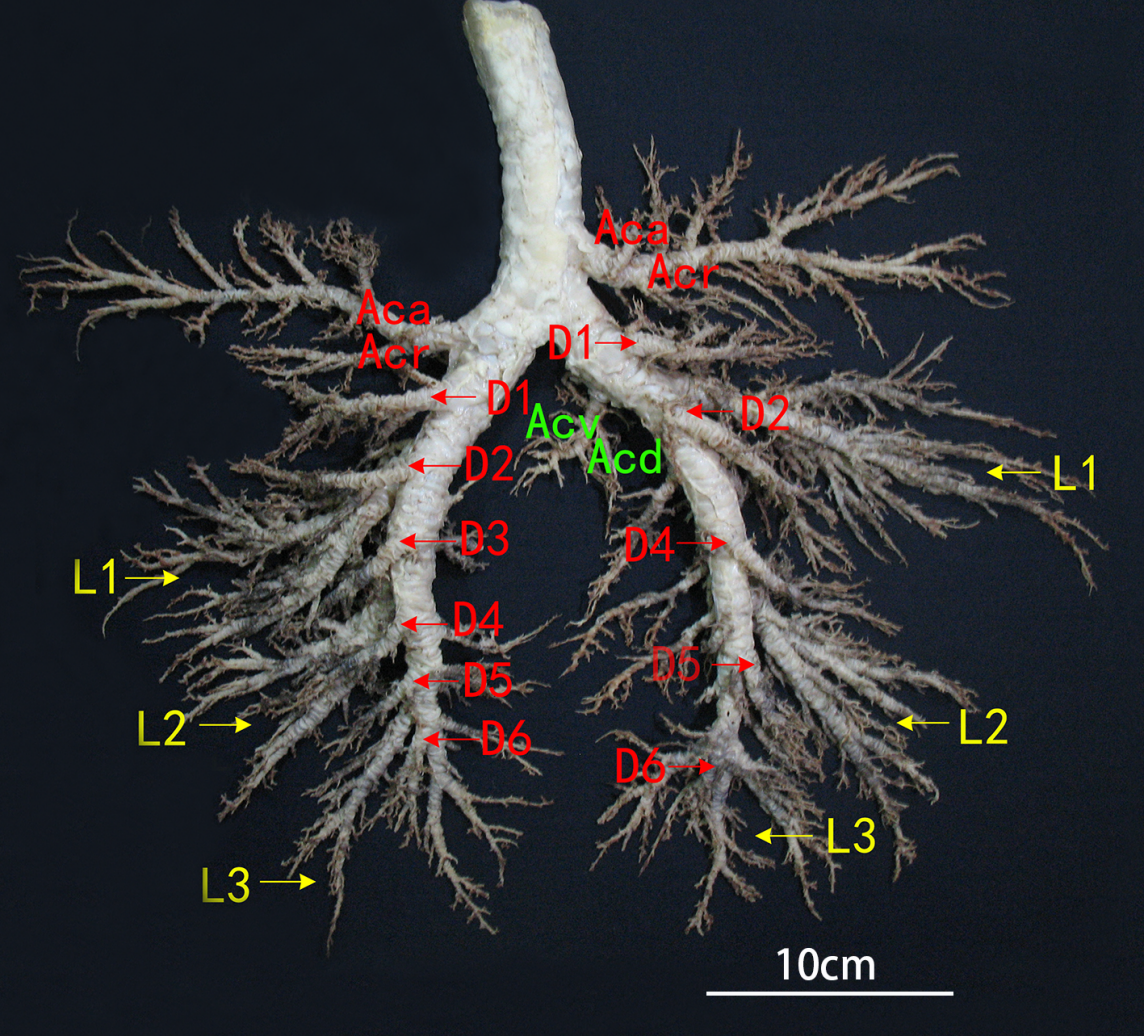
Dorsal view of the bronchial tree of Bactrian camels. Aca, Caudal segmental bronchi of the cranial lobe; Acr, Cranial segmental bronchi of the cranial lobe; Acv. Ventral segmental bronchi of the accessory lobe; Acd, Dorsal segmental bronchi of the accessory lobe; L1–L3, Lateral segmental bronchi from the first to third segment of the caudal lobe; D1–D6, Dorsal segmental bronchi from the first to sixth segment of the caudal lobe, and D3 was absent in the right lung. Photograph by Wanhong He.

The bronchial tree branches of Bactrian camels, with unequal dichotomies, included the dorsal, ventral, lateral and medial bronchiole systems. The trachea contained approximately 70 cartilaginous rings, with approximately three cartilaginous rings over the top of the tracheal bifurcation. The trachea had a tracheobronchial branch that formed the right cranial lobar bronchus, but the left cranial lobar bronchus was the first branch of the left main bronchus. In addition, there were cranial (Acr) and caudal (Aca) segmental bronchi in the cranial lobe; dorsal (D1-D6, and D3 was absent in right), ventral (V1-V5), medial (M3-M6) and lateral (L1-L3) segmental bronchi in the caudal lobe; and dorsal (Acd) and ventral (Acv) segmental bronchi in the accessory lobe. Moreover, depending on the bronchial lumen size and branch characteristics, Aca >Acr in the left cranial lobe; Aca <Acr in the right cranial lobe; and Acv >Acd in the right accessory lobe. In both the left and right caudal lobes, D and L were much larger than M and V, and L1 >L2 >L3, D2 >D1 >D3 >D4 >D5 >D6, V1 >V2 >V3, but V4, V5 and M3-M6 were all smaller branches, so there were unobvious differences among them (Figs. 3 and 4).

### The histological characteristics of Bactrian camel lungs

**Figure 5:**
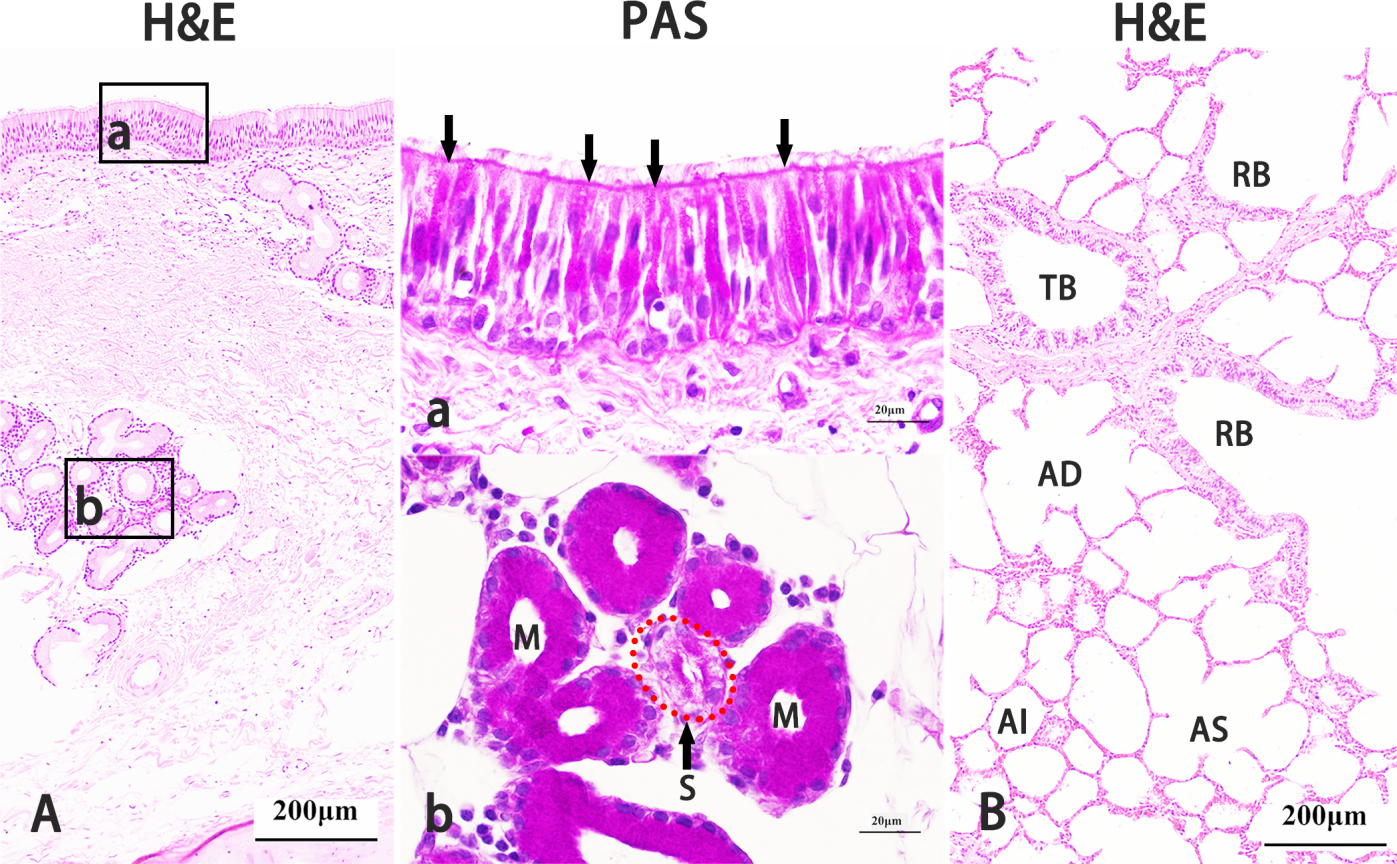
Histological characteristics of Bactrian camel lungs. The right main bronchial epithelium stained with haematoxylin and eosin (H & E) was a dense pseudostratified ciliated columnar epithelium (a), and there were numerous submucosal glands (b) between the epithelial basal lamina and cartilage. Original magnification: 100 × (A). The right main bronchial epithelium stained with periodic acid Schiff (PAS); goblet cells (sorrows) were well distributed in this epithelium. Original magnification: 1,000 × (a). Submucosal glands of the right main bronchi stained with PAS were composed of mucous glands (M) and serous glands (S, red dotted circle). Original magnification: 1,000 × (b). The lung respiratory portion was stained with H & E, there were few alveoli, and there were a number of alveolar tubes and alveoli sacs. TB, terminal bronchiole; RB, respiratory bronchiole; AD, alveolar ducts; AS, alveolar sacs; AI, alveoli. Original magnification: 100 × (B). Images by Wanhong He.

The Bactrian camel trachea was covered by dense pseudostratified ciliated columnar epithelium embedded with many goblet cells, and numerous submucosal glands (serous and mucous glands) were located between the epithelial basal lamina and the cartilage (Fig. 5A). However, as bronchial branches increased, the distribution of goblet cells and submucosal glands decreased and nearly disappeared in bronchioles with simple ciliated columnar epithelium. In the respiratory portion, there were few alveoli, and there were a number of alveolar tubes and alveoli sacs (Fig. 5B).

### The distributive and structural characteristics of BALT in Bactrian camels

**Figure 6:**
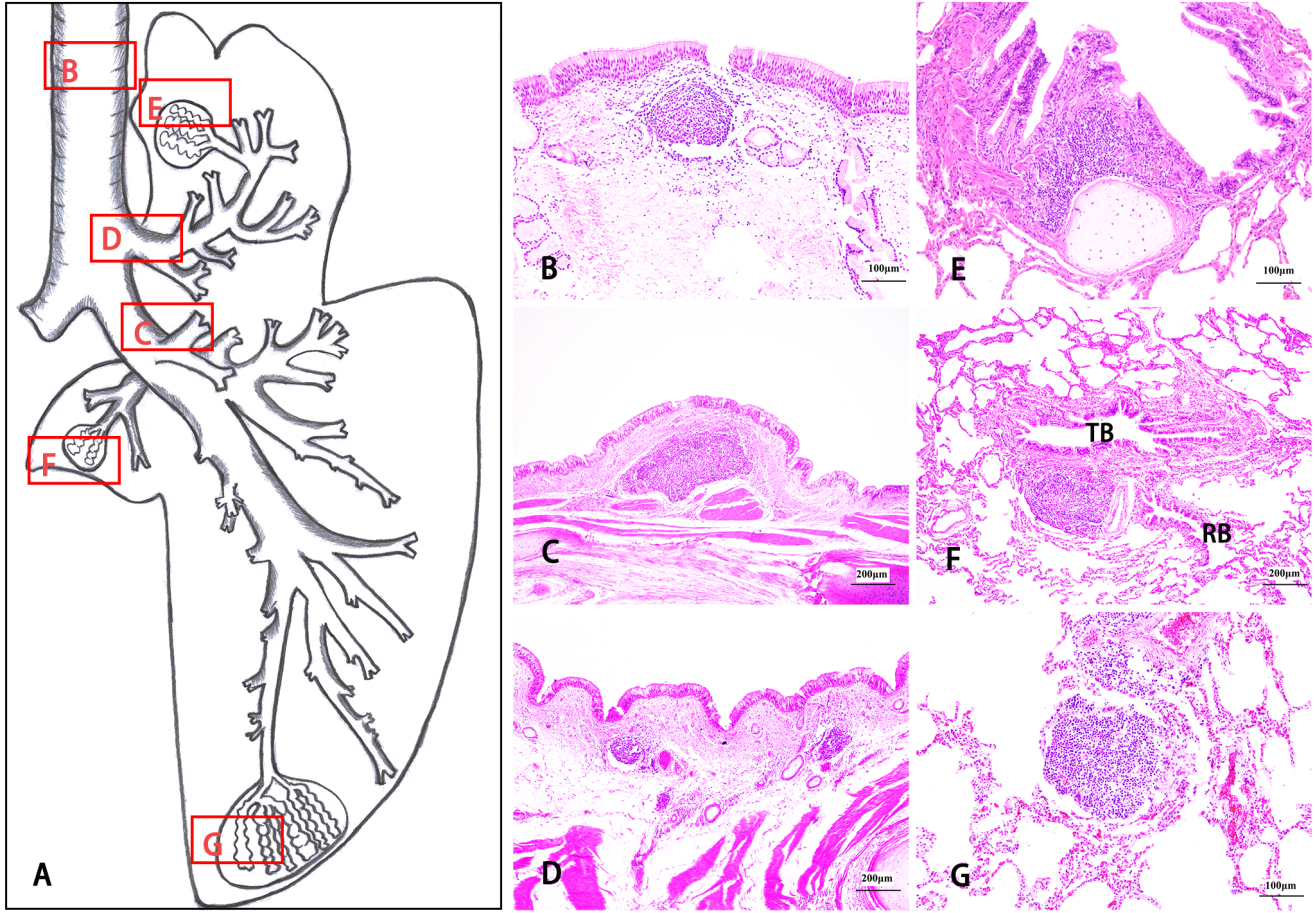
The morphological distribution characteristics of isolated lymphoid follicles (ILFs) in Bactrian camel lungs. Bronchus-associated lymphoid tissue (BALT) was scattered along the bronchial tree in the whole lung, but in the conducting portion, BALT was mainly located in the mucosa lamina propria (LP) and was still found in the submucosa, under the muscular layer, and around the submucosal glands and cartilage. ILFs are one type of BALT and exist from the trachea to the pulmonary mesenchyme. (A) is a schematic drawing of the right lung, including the cranial lobe, caudal lobe and accessory lobe, in Bactrian camels. (B–G) stained with haematoxylin and eosin (H & E), show the histological characteristics of ILFs in different locations: ILFs in the trachea LP. Original magnification: 200 × (B); ILFs in the submucosa of the first dorsal segmental bronchi (D1) of the caudal lobe. Original magnification: 100 × (C); ILFs in the submucosa around the mucosal glands of the cranial lobar bronchi. Original magnification: 100 × (D); ILFs in the bronchiole LP of the cranial lobe. Original magnification: 200 × (E); ILFs between the terminal bronchiole (TB) and the respiratory bronchiole (RB) in the accessory lobe. Original magnification: 100 × (F); ILFs in pulmonary mesenchyme of the caudal lobe. Original magnification: 200 × (G). Images by Wanhong He.

**Figure 7:**
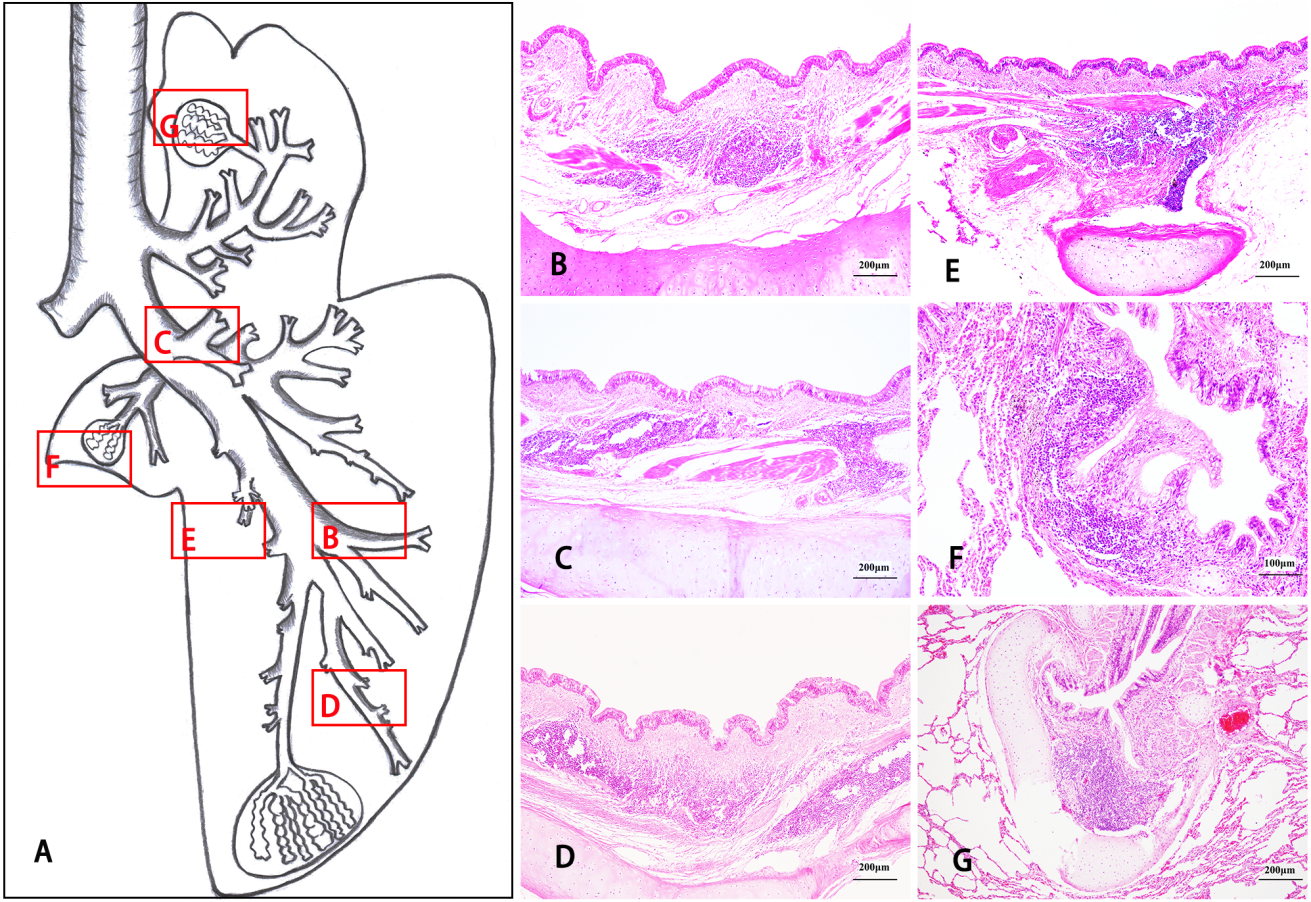
The morphological distribution characteristics of aggregates of lymphoid follicles in Bactrian camel lungs. The aggregates of lymphoid follicles (ALFs), the other type of BALT in Bactrian camels, were sporadically distributed in the segmental bronchi, small bronchi and bronchioles. However, in the conducting portion, ALFs are mainly located at the mucosa lamina propria (LP) and can be found in the submucosa, under the muscular layer, and around the submucosal glands and cartilage. Picture A is a schematic drawing of the right lung, including the cranial lobe, caudal lobe and accessory lobe, in Bactrian camels. (B–G) stained with haematoxylin and eosin (H & E), show the histological characteristics of ALFs in different locations: ALFs in the submucosa and under the muscular layer of the second lateral segmental bronchi (L2) of the caudal lobe. Original magnification: 100 × (B); ALFs in the submucosa and around the cartilage of the first dorsal segmental bronchi (D1) of the caudal lobe. Original magnification: 100 × (C); ALFs in the submucosa, under the muscular layer and around the cartilage of the third lateral segmental bronchi (L3) of the caudal lobe. Original magnification: 100 × (D); ALFs under the muscular layer and around the cartilage of the second ventral segmental bronchi (V2) of the caudal lobe. Original magnification: 100 × (E); ALFs in the bronchiole mucosa lamina propria (LP) of the accessory lobe. Original magnification: 200 × (F); ALFs in the small bronchi LP of the cranial lobe. Original magnification: 100 × (G). Images by Wanhong He.

**Figure 8:**
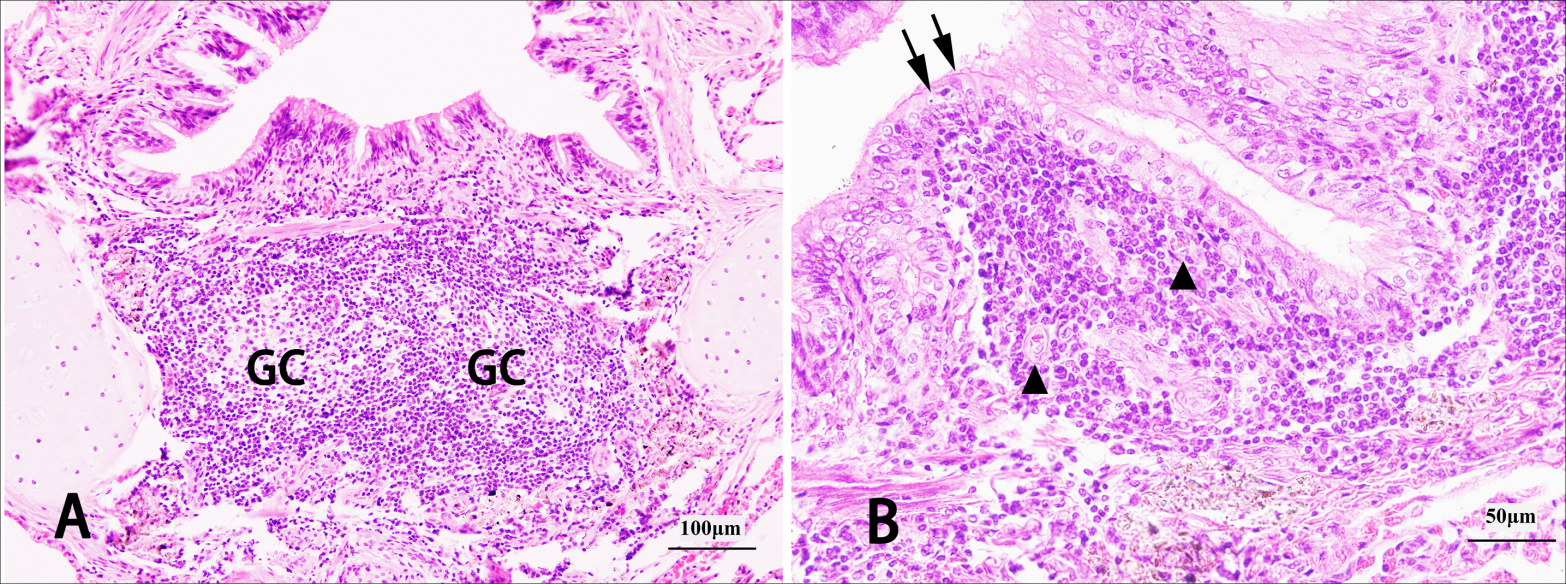
Structural characteristics of well-developed BALT in Bactrian camels. Germinal centres (GCs) in BALT, stained with haematoxylin and eosin (H & E). Original magnification: 200 × (A); obvious follicle-associated epithelium (FAE) (sorrow) and high endothelial venules (HEVs) (triangle) in BALT, stained with H & E. Original magnification: 400 × (B). Images by Wanhong He.

The isolated lymphoid follicles (ILFs) (Fig. 6) and aggregates of lymphoid follicles (ALFs) (Fig. 7) were two types of BALT, and germinal centres (GCs) (Fig. 8A), HEVs and follicle-associated epithelium (FAE) (Fig. 8B) were observed in some well-developed BALT. ILFs, as the chief type of BALT, spread from the trachea to the pulmonary mesenchyme, but sporadic ALFs were mainly distributed in segmental bronchi, small bronchi and bronchioles. Moreover, BALT was scattered along the bronchial tree in the entire lung of the Bactrian camel, including each branch of the dorsal, ventral, lateral and medial bronchiole systems, and density increased from the trachea to the lower graded branches (densest in the bronchiole) and then decreased, with the occasional location around the respiratory bronchioles or among the pulmonary mesenchyme. Amazingly, in the bronchial conducting portion, the majority of the BALT was located in the mucosa lamina propria (LP), but in the submucosa, under the muscular layer, and around the submucosal glands and cartilage, BALT could still be found (Figs. 6 and 7).

## Discussion

### The anatomical characteristics of lung and bronchial branches in Bactrian camels

Bactrian camel lung lobes include the left cranial lobe, left caudal lobe, right cranial lobe, right caudal lobe and right accessory lobe, and were similar to those of horses (Dong, 2009) but were less similar to those of other ruminants (Dong, 2009). According to biological evolution studies, the appearance of the diaphragm is the root reason for lung lobe occurrence because the lung lobe can promote the initiation of breathing activity, and the lobar number is positively associated with respiration intensity (Yu & Yang, 1958). Visibly, the lower lung lobe of Bactrian camels is beneficial for maintaining a low breathing rate (Chen, 2008), which helps camels adapt well in arid environments with low water consumption.

However, in the unequal dichotomous bronchial tree branches of Bactrian camels, approximately three cartilaginous rings were located over the top of the tracheal bifurcation, and there was a tracheobronchial branch forming the right cranial lobar bronchi. In fact, this tracheobronchial branch is absent in higher mammals and is found only in ruminants (cattle, yak, sheep) and pigs (Dong, 2009). It has been shown that the developmental degrees of the lung cranial and caudal lobes largely rely on the mammal’s respiratory patterns. For example, if abdominal breathing is dominant, the caudal lobe is more developed (cattle, sheep), but if the thoracic breathing is dominant, the cranial lobe is more developed (human, dog), and thoracic breathing has become increasingly predominant over the course of mammalian evolution (Yu & Yang, 1958). In addition, the angle between the tracheobronchial branch and trachea is too small to provide good ventilation (Hyde, Tyler & Plopper, 2007); therefore, with the increased demands of the cranial lobe in thoracic breathing, the tracheobronchial branch might be gradually degenerated and replaced by the first branch of the right main bronchus.

Finally, compared with humans (Nakakuki, 1975) and other animals (horse, cattle, pig) (Nakakuki, 1994a; Nakakuki, 1994b; Nakakuki, 1994c), the construction of the dorsal, ventral, lateral and medial bronchiole systems of Bactrian camels showed some species specificity, for example, the dorsal bronchiole system included D1 to D6, but D3 was absent in the right lung. However, interestingly, in horses (Nakakuki, 1994a), the ventral bronchiole system includes V3 to V5, but V4 is absent in the right lung; in cow (Nakakuki, 1994c), the medial bronchiole system includes M4 to M6, but only M6 in the right lung, and the ventral bronchiole system includes V3 to V5 in the left lung, but V2 to V4 in the right lung. Therefore, there were obvious differences in the construction of these four bronchiole systems in different mammals, but reasons are unclear at present.

Above all, the lesser lung lobar number, narrower cranial lobe and more developed caudal lobe of Bactrian camel lungs were similar to those in horses, but the bronchial tree with a tracheobronchial branch was more closely related to that of ruminants and was identical to the morphological and structural variation regularity of lungs with species evolution.

### The distributive and structural characteristics of BALT in Bactrian camels

BALT was mainly scattered in the bronchial conducting portion of Bactrian camels, and the density expressed increased from the trachea to the lower graded branches (densest in the bronchioles) and then decreased, with occasional distribution around the respiratory bronchioles or among the pulmonary mesenchyme. Many studies have shown that BALT distribution sites are inconsistent in different species. For example, BALT is located along the entire bronchial tree but mainly at the tracheal bifurcation in rabbits (Bienenstock et al., 1974); between bronchi and arteries in mice and rats (Breel et al., 1988; Gregson, Davey & Prentice, 1979); at small bronchi and bronchioles in Dromedary camels (Elhussieny et al., 2017) and pigs (Delventhal et al., 1992); and scattered in the total bronchial tree, but mainly in the lobar and segmental bronchi, in humans (Richmond et al., 1993). Therefore, the BALT distribution characteristics in Bactrian camels were similar to those of Dromedary camels and pigs but more closely related to humans. In fact, the bronchiole, a transitional structure with conducting and respiratory functions in the lung, is likened to a hinge; on the one hand, final filtration airflow goes down into respiratory portion, while on the other hand, it is the first barrier for gas exchange products coming back into the conducting portion. Additionally, the bronchiole itself is a susceptible place to induce pulmonary diseases (Crapo et al., 2000; Ryu, Myers & Swensen, 2003). As a result, BALT scattered along the entire bronchial tree was beneficial for rapidly inducing an immune response. Abundant BALT in bronchioles, as a final “checkpoint” in the surveillance, capture and recognition of the relative antigens before pulmonary exchange, could simultaneously maintain normal bronchiole function and reduce pulmonary disease incidence in Bactrian camels.

ILFs (the chief type) and ALFs were the two types of BALT in Bactrian camels. According to reports, these two types of BALT also exist in humans and the majority of animals (i.e., rabbits, mice, chickens, sheep, pigs and Dromedary camels) (Bienenstock et al., 1974; Delventhal et al., 1992; Elhussieny et al., 2017; Foo & Phipps, 2010). However, unexpectedly, in the conducting portion of Bactrian camels, BALT was mainly located at the LP but was still found in the submucosa, under the muscular layer, and around the submucosal glands and cartilage. Many studies demonstrate that BALT in the LP occurs by transmembrane antigenic stimulation (Moyron-Quiroz et al., 2004; Pabst & Tschernig, 2010), but if stimulating factors are gradually removed, the immune-induced function of BALT will decline, and BALT will eventually migrate from the LP to the submucosal layer (or deeper areas) and disappear (Hwang, Randall & Silva-Sanchez, 2016; Pabst & Tschernig, 2010). BALT in different areas of the bronchial mucosa of Bactrian camels might be closely related to their own developmental degeneration and antigen stimulation, which needs to be confirmed by further research.

## Conclusions

The distributive and morphological characteristics of BALT in Bactrian camel lungs were systematically observed and analysed in this study. The results demonstrated that the lung morphology of Bactrian camels was similar to that of horses, but the bronchial branches were more closely related to those of ruminants. These characteristics were in accordance with the morphological and structural variation regularity of lungs with species evolution. BALT was mainly scattered along the bronchial tree and bronchioles as the final “checkpoint” in surveillance, and the capture and recognition of the relative antigens before pulmonary exchange was the pivotal distribution position of BALT. However, BALT in different areas of the conducting portion might be at different developmental stages. Our study provided evidence for further insight into the mucosal immunomodulatory mechanism of BALT in the respiratory system of Bactrian camels.

## Additional Information and Declarations

### Data Availability

The following information was supplied regarding data availability: The raw data are included in the Figures.
